# Effects of Phthalate Esters (PAEs) on Cell Viability and Nrf2 of HepG2 and 3D-QSAR Studies

**DOI:** 10.3390/toxics9060134

**Published:** 2021-06-05

**Authors:** Huan Liu, Huiying Huang, Xueman Xiao, Zilin Zhao, Chunhong Liu

**Affiliations:** 1 College of Food Science, South China Agricultural University, Guangzhou 510642, China; liuhuanjzcd@126.com (H.L.); huozangqu@126.com (H.H.); xiaoxueman2017@163.com (X.X.); wzhaozilin@163.com (Z.Z.); 2 Guangdong Provincial Key Laboratory of Food Quality and Safety, Guangzhou 510642, China

**Keywords:** phthalate esters, Nrf2, 3D-QSAR, HepG2

## Abstract

Phthalate esters (PAEs) are a widespread environmental pollutant, and their ecological and environmental health risks have gradually attracted attention. To reveal the toxicity characteristics of these compounds, ten PAEs were selected as research objects to establish a cell model. CCK-8 was used to determine cell viability, Western blots were used to determine the content of Nrf_2_ in HepG2, and the LD_50_ collected for the 13 PAEs administered to rats. On this basis, 3D-QSAR models of IC_50_, LD_50_ and Nrf2 were established. The experimental results showed that as the time of PAEs exposure increased (24, 48 and 72 h), cell viability gradually decreased. The test concentration (62.5 /125/250 μM) of PAEs exposed for 48 h could significantly increase the content of Nrf2, and the 1000 μM PAEs could inhibit the content of Nrf2. The model is relatively stable and predicts well that the introduction of large and hydrophobic groups may significantly affect the toxic effects of PAEs on cells. The present study provided a potential tool for predicting the LD50 and Nrf2 of new PAEs, and provide a reference for the design of new less toxic PAEs in the future.

## 1. Introduction

Phthalate esters (PAEs) are mainly used as plasticisers that can be employed in various chemical products to promote processing and product flexibility [1,2]. Since their first application in industrial products in the 1920s [3], their global production and consumption have increased to more than 6 million tons [4]. Historically, the use of phthalic acid (2-ethylhexyl) ester (DEHP) in polyvinyl chloride (PVC) was very common, and DEHP was later replaced by didecyl phthalate (DIDP) and diisononyl phthalate (DINP) [5]. Low molecular weight phthalates including dibutyl phthalate (DBP), diethyl phthalate (DEP), and dimethyl phthalate (DMP), were mostly used in personal care products, pesticides, glue, and paint solvents [6,7]. However, due to the weak binding force between phthalates and substrates, the PAEs could be gradually released from the product to the environment during production, use, disposal and recycle [8,9], leading to frequent and undesirable human contact.

The persistent existence of PAEs in the environment and their toxicity has aroused widespread concern in society. Many experiments have revealed that some PAEs could cause developmental toxicity, reproductive toxicity, hepatotoxicity, neurotoxicity, and even carcinogenicity, teratogenicity, and mutagenicity [10]. For example, in a study compared with the workers who were not exposed to high levels of DEHP and DBP, workers exposed to high levels DEHP and DBP had significantly lower free testosterone [11]. In another study, exposure of pregnant women to various PAEs led to a shortened anal-genital distance in male infants [12].

Hepatotoxicity is also commonly observed as systemic toxicity in rodents exposed to PAEs. The liver is the main target of PAEs because it is the main detoxification site for the decomposition of PAEs in the body. It has been reported that some PAEs caused peroxisome proliferation and liver tumors through peroxisome proliferation factor activated receptors (PPARs) [13]. Similarly, DEHP could disrupt the homeostasis of thyroid hormones by inducing liver enzymes, leading to liver edema [14]. In addition, high-fat diets exposed to DEHP can cause various degrees of non-alcoholic fatty liver in mice [15].

Furthermore, hepatotoxicity caused by PAEs is closely related to oxidative stress, and nuclear factor erythroid 2 related factor 2 (NRF2) is a nuclear transcription factor that regulates oxidative stress [16], which plays a crucial role in liver detoxification. Therefore, the impact of PAEs on Nrf2 has been directly related to the liver toxicity, with the toxicity of PAEs varying greatly due to their side chains and the type of analogues [17]. Considering the complexity, three-dimensional quantitative structure-activity relationship (3D-QSAR) model was introduced to explore the toxic effects of different PAEs. The field of quantitative structure-activity relationship (QSAR) research in medicinal chemistry has been greatly developed since the 1970s for the quantitative studies om the mathematical relationship between the chemical structure and pharmacological activity or other properties of a series of compounds [18].

Therefore, the aim of this study was to develop a 3D-QSAR model based on the investigation of the effects of PEs on Nrf2, IC50 in human hepatocyte (HepG2) cells, in the hope of predicting the toxicity of new analogues and providing a reference for the design of less toxic PAEs in the future.

## 2. Materials and Methods

### 2.1. Cell Culture

Human hepatocyte HepG2 was purchased from Procell (Wuhan, China) and cultured in MEM (Procell) complete medium supplemented with 10% fetal bovine serum (FBS), 1% penicillin streptomycin (pen-strep: 10,000 U/mL) and 1% L-glutamine. Cells were maintained in a 37 °C, 5% CO_2_, fully humidified incubator and passed twice weekly.

### 2.2. Cell Viability

To evaluate the cytotoxicity of DEHP and DMP on the cells, HepG2 cells were cultured in a 96-well plate at a density of 3 × 103 cells/well. After 24 h incubation, cells were taken at the logarithmic stage and treated with DEHP, dihexyl phthalate (DHXP), di-*n*-octyl phthalate (DNOP), dinonyl phthalate (DNP), bis(2-methoxyethyl) phthalate (DMEP), DEP, and DIDP (62.5, 125, 250, 500 and 1000 μM), or DMSO (solvent control, final concentration <0.1%) (purchased from Sigma, St. Louis, MO, USA). After 24, 48 and 72 h exposure, the viability was measured using CCK-8 cell viability assay kit (Nanjing Jiancheng Bioengineering Institute, Nanjing, China). After different treatments, 10 μL CCK-8 solution was added into the culture and incubated at 37 °C for 3 h. The generated luminescent signal was captured on a Microplate Reader (Multiskan FC, Thermo, Waltham, MA, USA). The results were expressed as percentage of control, and each value was presented as the mean ± SD of at least three independent experiments.

### 2.3. Western Blot Analysis

Cells were inoculated in six-well plates for 24 h and then treated with DEHP, DEP, DMP, DMEP, DHXP, DNOP, DBP or DMSO for 48 h. After treatment, RIPA lysate (50 mM Tris (pH 7.4), 150 mM NaCl, 1% Triton X-100, 1% sodium deoxycholate, 0.1% SDS, (Beyotime Biotechnology, Haimen, China) was added to lyse for 30 min to extract the total protein. Then, the cell lysate was centrifuged at 12,000× *g* for 5 min, and the supernatant was collected. Protein concentration in the extract was determined with the Bicinchoninic Acid assay (Beyotime Biotechnology). Equal amount of protein samples (15 g/lane) were subjected to 10% sodium dodecyl sulfate polyacrylamide gel electrophoresis (SDS-PAGE) and then adsorbed on polyvinylidene fluoride (PVDF) membranes (Millipore Corporation, Bedford, MA, USA), which were sealed by 1.5 h in 5% (*w*/*v*) skim milk. Afterwards, the membranes were incubated with primary antibodies against Nrf1(1:2000, abcam, Cambridge, UK) and tubulin alpha (1:6000, Affinity Biosciences, Changzhou, China), followed by a horseradish peroxidase- (HRP-) conjugated with goat anti-rabbit IgG antibody (Nanjing Jiancheng Bioengineering Institute, Nanjing, China) diluted to 1:10,000 at room temperature for 1 h. Finally, the protein bands on the membrane were visualized using ECL western blot detection reagent (BioSharp, Technology Inc., Shanghai, China) and quantified using ImageJ software (National Institutes of Health, Bethesda, MD, USA).

### 2.4. D-QSAR

#### 2.4.1. CoMFA and CoMSIA for LD_50_ of PAEs

In the quantitative structure-activity relationship modeling, the biological activity value LD_50_ (collected from PubChem) was calculated in mg/kg, as shown in Table 1. CoMFA and CoMSIA methods were used for 3D-QSAR analysis [19], and all operations were completed with SYBYL2.1.1 software module. The molecular dynamics program Minimize was used to optimize the energy of all compounds to obtain their lowest energy conformation. In the optimization process, the Tripos force field, Powell energy gradient and Gasteiger-Huckel charge were applied, and the termination conditions and max iteration conditions were respectively 0.005 kcal/(mol × A) and 1000. Other parameters were default values. DMP was used as the template molecule, and all molecules were superimposed using the Align Database method. The training set was DMP, DBP, DIBP, DHXP, DNOP, diisooctyl phthalate (DIOP), DEHP, DNP, 1,2-benzenedicarboxylic acid,1-decyl 2-octyl ester (nDNOP), diallyl phthalate (DAP), and the test set was DMEP, DEP, and DIDP (using the method of random assignment to determine the training set and test set). Statistical analysis adopted partial least squares analysis (PLS) to establish the model, and then cross-validation was carried out by leave-one-out (LOO) methodology.

#### 2.4.2. CoMFA and CoMSIA for IC_50_ of PAEs

HepG2 cells were used as experimental objects to explore the effects of PAEs exposure for 24 h, 48 h and 72 h on cell viability, and the IC_50_ of PAEs exposure for 72 h on HepG2 cells was calculated as model biological activity data. Using DEHP, DMEP, DNP, DNOP, DIDP as the training set and DPHP, DHXP as the test set to establish CoMFA and CoMSIA models. The molecular modeling method and parameters were as elaborated in Section 2.4.1.

#### 2.4.3. CoMSIA for Nrf2 of PAEs

Western blots were used to determine the content of Nrf2, a key antioxidant protein, in HepG2 cells exposed to PAEs for 48 h, which was used as the model biological activity data. Taking DEHP, DMP, DBP, DNOP, DMEP as the training set, and DEP, DHXP as the test set to establish the CoMSIA model. The molecular modeling method and parameters were as elaborated in Section 2.4.1.

### 2.5. Applicability Domain

We used a Leverge-based approach to calculate the range of use of the model and represented it visually using Williams diagrams. Here leverage denotes the distance between the value of the *ith* observation *x* and all *X* and was calculated as:

*h_i_* = *x_i_* (*X^T^ X*)^−1^*x_i_^T^*

where *x^i^* is the vector of variables for compound *i* to be tested and *X* is the matrix of independent variables for the training set. The general threshold for whether a sample is abnormal or not is set to h* = 3 k/n, where k is the variable term and n is the number of samples. If the leverage value of a compound is higher than h*, it is considered an *X* outlier.

### 2.6. Statistical Validation

To test the fitting quality and prediction capability of continuous QSAR model, we used Model Acceptance Criteria in Enalos+ KNIME nodes [20]. According to the criteria proposed by Tropsha A, the following conditions must be satisfied:

R^2^ > 0.6; Rcvext^2^ > 0.5; (R^2^–R0^2^)/R^2^ < 0.1; (R^2^–R’0^2^)/R^2^ < 0.1; abs(R0^2^–R’0^2^) < 0.3; 0.85 ≤ k ≤ 1.15; 0.85 ≤ k’ ≤ 1.15

where R^2^ is the correlation coefficient between the predicted and observed activities, Rcvext^2^ the external cross validation, R0^2^ the coefficient of determination: predicted versus observed activities, R’0^2^ the coefficient of determination: observed versus predicted activities, k = slope: predicted versus observed activities regression lines through the origin and k’ = slope: observed versus predicted activities regression lines through the origin.

### 2.7. Statistical Analysis

All raw data were collated in a Microsoft Excel database, whilst SPSS 18.0 (SPSS Inc., Chicago, IL, USA) and GraphPad Prism 8.0 (GraphPad Inc., La Jolla, CA, USA) were used for statistical analysis. Experimental data were expressed as means ± standard deviation (means ± SD). Differences among groups were compared with one-way analysis of variance with the significant difference acceptance at *p* < 0.05, and the extremely significant difference acceptance at *p* < 0.01 confidence levels, respectively.

## 3. Results and Discussion

### 3.1. Cell Viability

PAEs are a class of exogenous compounds and HepG2 cells are derived from human hepatocytes, which have similar metabolic functions to hepatocytes and are a common cell model for studying the toxicity of exogenous compounds, hepatotoxicity and mitochondrial toxicity. Meanwhile, HepG2 is probably considered to be one of the first (one of the first) hepatocyte lines to be isolated and widely used. It is relatively inexpensive to culture, relatively well maintained and readily available. Therefore, we chose HepG2 cells as the subject of our study. The present study utilized CCK-8 to determine the effects of seven PAEs exposed for different times (24, 48 and 72 h) on the viability of HepG2 cells. 

**Figure 1:**
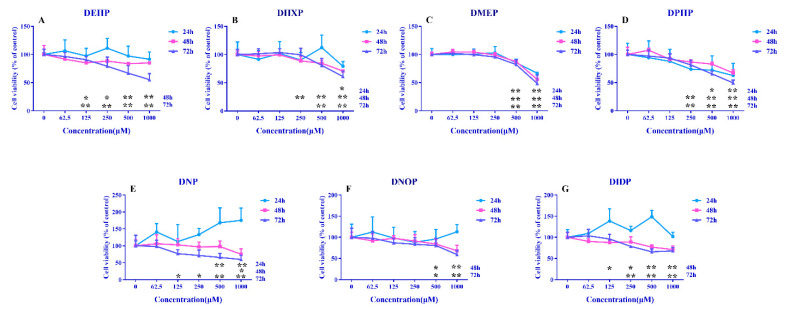
The influence of PAEs on cell viability. Cells were treated with 62.5, 125, 250, 500 and 1000 μM PAEs for 24 h, 48 h and 72 h. (**A**): DEHP, (**B**): DHXP; (**C**): DMEP; (**D**): DPHP; (**E**): DNP; (**F**): DNOP; (**G**): DIDP. Data are expressed as mean ± SD, n = 6. * *p* < 0.05, ** *p* < 0.01 versus the control group.

The results presented in Figure 1 show that the cell viability decreased significantly (*p* < 0.05), particularly at 72 h of exposure for all the tested PAEs, except DMP. PAEs had a certain accumulation. With the extension of exposure time, the accumulation of PAEs in cells increased, so the toxicity increased significantly. Further, the results in this study are agreement with that of previous ones wherein DEHP was observed to induce toxicity in different types of cells by severely inhibiting the proliferation of Hep3B [21].

### 3.2. Nrf2 Protein Content

Nrf2, a ubiquitous transcription factor that could mediate and regulate the expression of more than 200 antioxidant enzymes and cytoprotective proteins [22] was used as the main mediator in this study. Previous studies identified the Nrf2 signalling pathway as the main host defense pathway in the body. This transcription factor, Nrf2 enters the nucleus and interacts with the downstream HO-1 protein to activate the oxidative stress pathway to protect the cell that is not coerced when the intracellular ROS increases [23]. 

**Figure 2:**
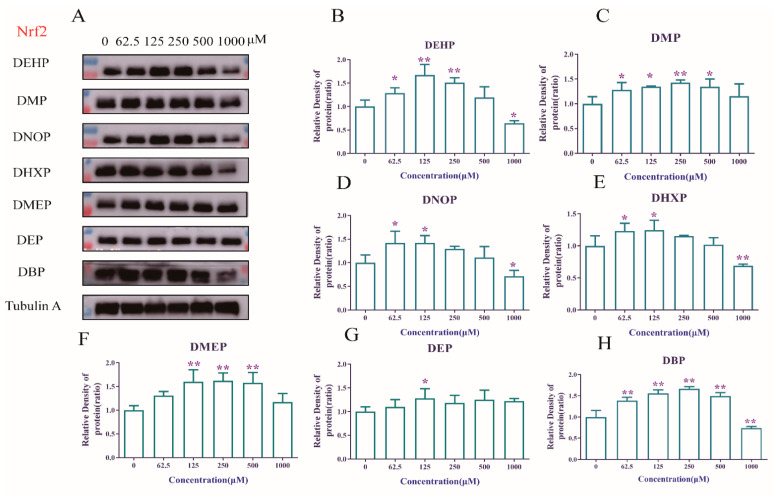
The influence of PAEs on cell Nrf2 content. Cells were treated with 62.5, 125, 250, 500 and 1000 μM PAEs for 48 h. (**A**): Western blot strips of PAEs; (**B**): DEHP, (**C**): DMP; (**D**): DNOP; (**E**): DHXP; (**F**): DMEP; (**G**): DEP; (**H**): DBP. Data are expressed as mean ± SD, *n* = 3. * *p* < 0.05, ** *p* < 0.01 versus the control group.

It is observed that the inhibitory effect of Nrf2 enhances the lipid peroxidation of the liver, which indicates that the activation of Nrf2 plays an important role in the antioxidant defense mechanism of the liver [24]. However, some recent studies have shown that DEHP exposure caused oxidative stress by interfering with the transcripts of the Nrf2 signaling pathway and its downstream genes [25]. After DEHP exposure, INS-1 cells produced a large amount of ROS leading to the imbalance in the NRF2-dependent antioxidant defense protection [26]. In the present study, Nrf2 was observed to have undergone similar changes (Figure 2). After 48 h of exposure to DEHP, DMP, etc., Nrf2 was inhibited in the cells of the high-dose group, which causes a certain degree of oxidative stress. However, low concentration PAEs in the test concentration increased the expression of Nrf2 after 48 h, the result was accord with other studies which revealed that DEHP induced testicular toxicity through oxidative stress and up-regulated Nrf2 expression [27]. Considered together, under the stimulation of low concentration PAEs in the test concentration, the Nrf2 protein content increased, indicating that HepG2 cells can trigger a defense mechanism to combat the toxicity of low concentration PAEs in the test concentration [28]. Meanwhile with the increased dose and the exposure time, the Nrf2 signaling pathway was inhibited, and subsequently the production of free radicals and the antioxidant defense system were out of balance, making the cells enter a state of oxidative stress. However, due to the complexity of the branched chains of PAEs, the changes were also different, and even some PAEs did not cause a significant decrease in Nrf2.

### 3.3. LD_50_ 3D-QSAR

**Figure 3:**
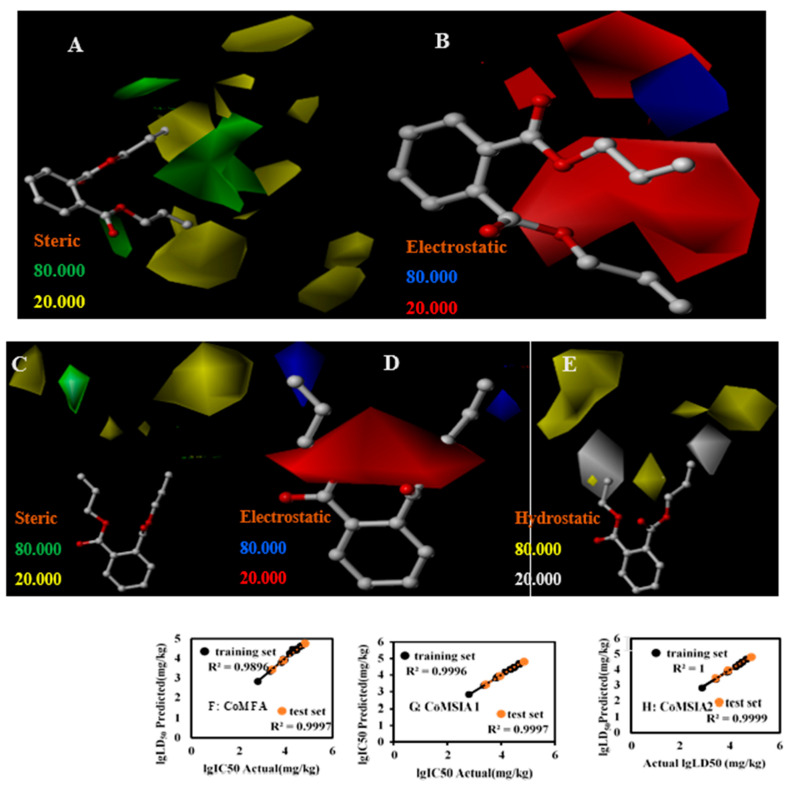
DAP’s three-dimensional contour maps of steric field (**A**), the electrostatic field (**B**) in the CoMFA model and steric field (**C**), the electrostatic field (**D**), hydrophobic field (**E**) in the CoMSIA2 model. The relation schema between predicted values and experimental values on PAEs’ lgLD50 ((**F**): CoMFA; (**G**): CoMSIA1; (**H**): CoMSAI2). Structural superimposition of PAEs(I).

Additionally, a 3D-QSAR method was used to quantitatively analyze the toxic effects of PAEs on rats. The results obtained, reported in Table 2, show that both the CoMFA model and CoMSIA1 model have good predictive ability with cross-validation coefficient (q2) greater than 0.50 [29,30] with values equal to 0.522 and 0.621, respectively. Other performance metrices evaluated were the standard error of estimate and Fischer’s value which were 0.067 and 192.02 and 0.015 and 1528.55 for CoMFA and CoMSIA1 models respectively. Furthermore, the non-cross validation coefficients were all greater than 0.90, indicating strong adaptability and robustness of the developed models [31,32]. The correlation between the predicted value and the actual value of training set and test set r2 were 0.9896 and 0.9997 in CoMFA (Figure 3F), and correlation between the predicted value and the actual value of training set and test set r2 were 0.9996 and 0.9997 in CoMSIA1, indicating that the model had good predictive ability. In addition, the contribution rates of the steric field (S) and the electrostatic field (E) in the CoMFA model were 58.4% and 41.6%, respectively. The contributions of the S, E, hydrophobic field (H), hydrogen bond donor field (D) and acceptor field (A) in the CoMSIA1 model were 18.8%, 26.6%, 50.8%, 3.8% and 0 respectively (Table 2). As can be observed from the results, contrastingly the structure-activity relationship between the hydrogen bond donor field and acceptor field was extremely small. Hence a CoMSIA2 model was established without considering the D and A fields. Subsequently, this approach was observed to improve the predictive performance with a q2 of 0.631 and r2 of 1 (Table 2). In this case, the contribution rates of S, E and H were 19.3%, 28.4% and 52.3% (Table 2), respectively. Further as can be observed from the figure the r2 of the model was 1.000 and 0.999 for the training and test sets, respectively.

In the CoMFA, CoMSIA1 and CoMSIA2 models evaluated in this study, the contribution rate of the S and the H accounted for 58.4%, 69.6%, and 71.6% (Table 2), respectively, suggesting that the non-specific reactions account for a larger proportion in the toxic effects of PAEs. The results of this study were consistent with other QSAR study on PAE biotoxicity and flammability which has shown that the hydrophobicity of molecules was an important parameter to characterize the toxicity of compounds [33]. This study also tried to apply the CoMSIA method to include the hydrogen bond donor and acceptor properties of the molecule. It was found that the above two parameters contributed very little to the structure-activity relationship, further confirming that non-specific reactions accounted for a relatively large proportion. However, whether it was CoMFA or CoMSIA1 and CoMSIA2 models, the electrostatic field had a greater contribution, accounting for 41.6%, 26.6% and 28.4% (Table 2), respectively. The QSAR model indicates that after PAEs enter cells, there may be electrostatic interactions between ester bonds, which would cause toxic effects through biochemical reactions.

The contour maps of CoMFA and CoMSIA models could also be used to explore the toxicity mechanism of compounds to a certain extent [34,35]. In the CoMFA model, two red areas appeared above the ether group of the electrostatic field equipotential diagram (shown in Figure 3B), and one red area appeared above the carbonyl group. These appearances indicate the introduction of negatively charged groups at the positions of the ether and the carbonyl groups, respectively which were responsible for the decreased toxicity of the compound. This effect was significant at the ether group wherein a significant electrostatic interaction with the possible acceptor was observed. In fact, in the stereo field equipotential diagram in Figure 3A, 12 yellow areas at the double bond positions on both sides of the DAP were observed. These regions indicated the introduction of large groups at the double bonds of the positively charged groups which increased the compound toxicity. As for the large groups introduced at the middle position of the double bonds on both sides and the right carbonyl group might have decreased the compound toxicity (Figure 3A). Similar additions on the bonds and equipotential results of the electrostatic fields were observed for the CoMSIA1 model as well. In fact, the presence of white and yellow areas on the side chains in the hydrophobic field (Figure 3E) confirm the influence of bond additions and electrostatic fields on the side chains and subsequent toxicity.

### 3.4. IC_50_ 3D-QSAR

**Figure 4:**
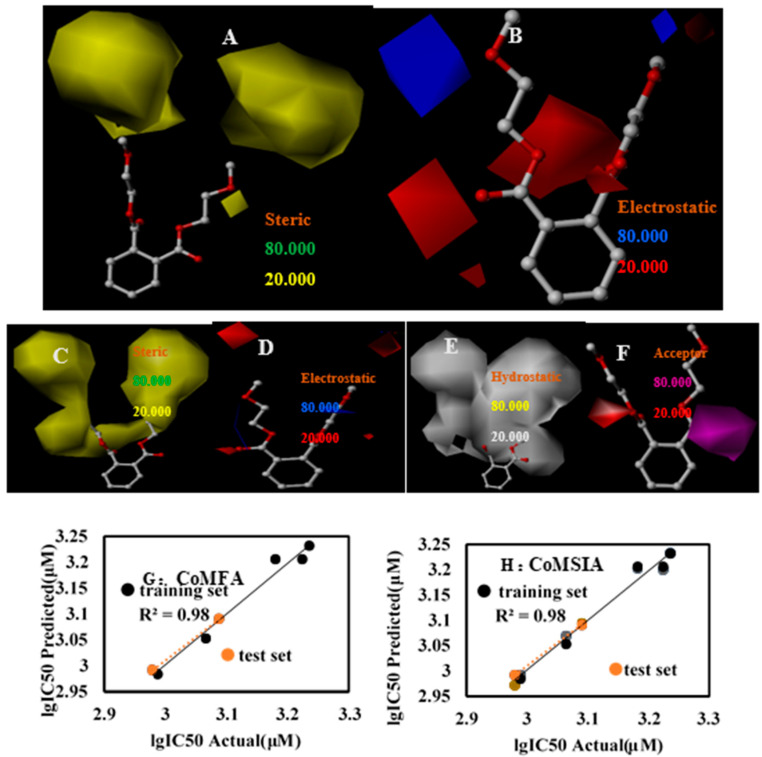
DMEP’s three-dimensional contour maps of steric field (**A**), the electrostatic field (**B**) in the CoMFA model and steric field (**C**), the electrostatic field (**D**), hydrophobic field (**E**), Acceptor field (**F**) in the CoMSIA model (**G**): CoMFA; (**H**): CoMSIA.

To further explore the toxic effects of PAEs, an in vitro cell experiment to determine the 50% lethal dose of PAEs to HepG2 cells was established. The experimental and model predicted values of 72 h-lgIC50 and the difference between them are detailed in Table 3. On the basis of the results, a CoMFA model and a CoMSIA model were built which respectively had a q^2^ of 0.69 and 0.687(Table 2), and r^2^ of 0.980 and 0.926 (Table 2). The non-cross validation coefficients were all greater than 0.9, indicating that the model was relatively stable. The training set correlation r^2^ between the predicted value and the actual value in CoMFA and CoMSIA were all 0.98 (Figure 4G,H), respectively, indicating that the model had good predictive ability. In addition, the contribution rates of S and E in the CoMFA model were 79% and 21% (Table 2), respectively. The contribution rates of S, E, H, D, and A in the CoMSIA1 model were 20.5%, 22.3%, 36.1%, 0 and 21.2% (Table 2), respectively. Like the 3D-QSAR model of LD_50_, the contribution rates of S and H were relatively high, especially the contribution rate of S in the CoMFA model reached 79% (Table 2), which has shown that non-specific reactions accounted for a relatively large proportion with a high probability of charge transfer.

From the three-position equipotential map of the CoMFA model, we found that the yellow area covered both sides of the DMEP molecule (Figure 4A), indicating that the introduction of large groups would enhance the activity of the molecule and lead to a decrease in IC_50_. The equipotential diagram in the electrostatic field shows that adding negatively charged groups near the carbonyl group and the ether group below could decrease the biological activity of the molecule (Figure 4B), thereby enhancing the biological toxicity of the molecule, whereas the introduction of negatively charged groups near the second ether group would cause the opposite effect. The position of the CoMSIA model was like that of the electrostatic field equipotential diagram, except that there were two red regions at the end of the side chain in the electrostatic field equipotential diagram, which show that the introduction of negatively charged groups was not beneficial to the biological activity.

In the hydrophobic field equipotential diagram with the largest proportion (Figure 4D), we were surprised to find that the white area was very large, almost covering the entire side chain, which indicated that the increase of hydrophilic groups in the side chain is closely related to the IC50 of PAEs on HepG2 cells, and this also confirmed the longer the fatty acid chain of PAEs, the lower IC_50_. In the hydrogen bond acceptor field (Figure 4F), we observed a red area on the left side of the ether group of DAP, indicating that adding hydrogen bond acceptors could increase molecular activity, but the carbonyl position on the right had a purple area, which had the opposite effect to the hydrogen bond acceptor on the left.

### 3.5. Nrf2 3D-QSAR

After exploring the effect of PAEs on the cell viability of the HepG2 cells, the mechanism was analyzed. The content of Nrf2, a key antioxidant protein, was determined, and a 3D-QSAR model of the effect of PAEs on Nrf2 was established. On this basis, we can predict the content of Nrf2 protein in HepG2 cells with different PAEs, and then understand the effect of PAEs on Nrf2 protein. On this basis, we established CoMSIA model, where q^2^ was 0.512 and r^2^ was 0.966 (Table 2).

**Figure 5:**
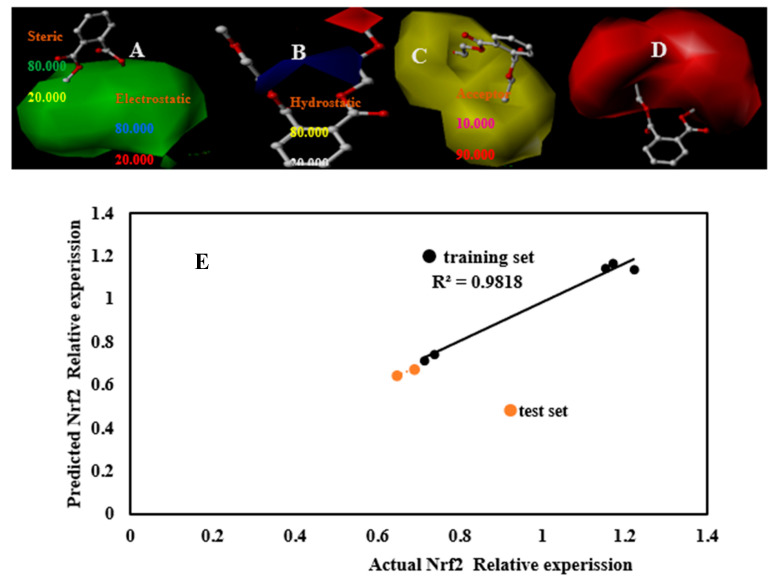
DMEP’s three-dimensional contour maps of steric field (**A**), the electrostatic field (**B**), hydrophobic field (**C**), Acceptor field (**D**) in the CoMSIA model. The relation schema between predicted values and experimental values on Nrf2 content ((**E**): CoMFA).

The non-cross validation coefficients were all greater than 0.9, indicating that the model was relatively stable. The training set correlation r^2^ between the predicted value and the actual value was 0.9818 (Figure 5E), indicating that the model has good predictive ability. In addition, the contribution rates of S, E, H, D and A in the CoMSIA1 model were 23.2%, 11.8%, 51.9%, 0 and 13.1% (Table 2), respectively.

The experimental and model predicted values of Nrf2 and the difference between them are detailed in Table 4. Like the 3D-QSAR model of LD_50_ and IC_50_, the contribution rates of S and H were relatively high, especially the contribution rate of H in the CoMFA model reaches 51.9% (Table 2), which has shown that non-specific reactions accounted for a relatively large proportion with probability of charge transfer.

Taking DMEP as an example, we found that the green area, yellow area and red area in the steric field, hydrophobic field, and hydrogen bond acceptor field were very large (Figure 5A,C,D). The more hydrogen bond acceptors on the branched chains of PAEs, the fewer large groups and the more hydrophilic groups, the more pronounced the decrease in Nrf2 content in HepG2 cells (Figure 5A,C,D). Adding negatively charged groups on the right-side branch in the electrostatic field equipotential diagram could increase the Nrf2 content, while adding positively charged groups near the carbonyl group could increase the Nrf2 content (Figure 5B).

Molecular-level toxic action mechanisms are generally divided into two categories: specific type and non-specific type; among them, the specific type refers to the presence of reactive substituent groups in the molecular structure of compounds, which were biochemically related to biological receptor molecules such as enzymes and proteins, which involved static electricity. Non-specific mechanism meant that molecules with similar structures could produce biological reactions with similar properties, and the reaction process was less dependent on special chemical structures. Organic matter enters the organism playing a role through the interaction with biofilms. The main controlling factor was the distribution ratio of chemical molecules in the organism and the water phase, related to the molecule’s hydrophobicity [36]. The addition of hydrophobic groups makes PAEs easier to enter cells. Generally speaking, the hydrophobicity of a molecule was closely related to the stereo field. Our results found that the changes in LD_50_, IC_50_ or Nrf2 were closely related to the hydrophobic field and the steric field, and the proportions were very large with the results like previous studies [33]. In fact, the hydrophobicity and volume of the groups had the most obvious effect on toxicity towards the HepG2 cells and Nrf2 expression. However, it is worth considering that the results for IC_50_ and Nrf2 were not similar, and that this opposite result may be due to the duration of exposure to PAEs However, it is worth considering that the IC_50_ results were not similar to those of Nrf2. This generalization of the result can help in predicting the toxicity of PAEs on the HepG2 cells and Nrf2 expression based on the sidechain and branch length of the PAEs without the necessity of experimentation.

### 3.6. Applicability Domain

**Figure 6:**
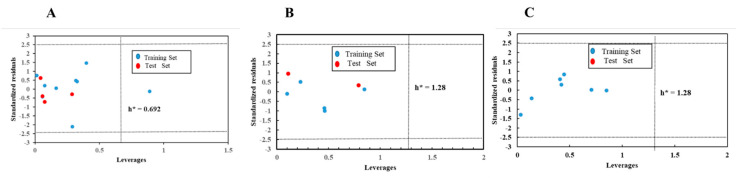
Williams plot for verifying the applicability domain of the model. (**A**): LD_50_; (**B**): IC_50_; (**C**): Nrf2.

We used normalised residuals and levers, visualised in Williams plots with training and validation sets, to evaluate AD. As shown in Figure 6, the horizontal and vertical dashed lines indicate the boundaries of normal values. The horizontal dashed lines were the Y (i.e., normalised residual) outliers, which were set ±2.5. An unknown compound was characterised as an outlier if its normalised residual exceeded the threshold value of 2.5. The vertical dashed line made the X (i.e., leverage) outliers, warning of leverage h* = 0.692 (A) or 1.28 (A, B). Figure 6A shows that all compounds were within the AD range except for DMEP (hi > h*) in the training set. Although methane shown high leverage, it has small normalised residuals, suggesting that it is a “good high leverage point” [37].

### 3.7. Statistical Validation

**Figure 7:**
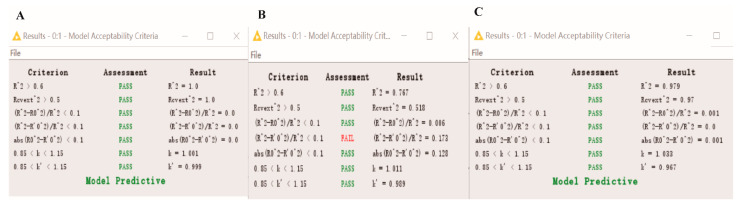
Enalos Model Acceptability Criteria KNIME node screenshot. (**A**): LD_50_; (**B**): IC_50_; (**C**): Nrf2.

The results of the test are shown in Figure 7. The predicted pLD_50_, IC_50_ and Nrf2 values correlated with the observed values within the tolerable error range. The values for the external R^2^ test were 1 (LD_50_), 0.767 (IC_50_) and 0.979 (Nrf2), supporting a good correlation between the predicted and observed values. However, one of the Tropsha’s test results for IC_50_ did not pass, indicating that the model for IC_50_ has some shortcomings in terms of predictive ability.

The Y-randomisation test is used to assess the robustness of the QSAR model. However, the small amount of data in our study makes it difficult to apply the Y-randomisation test. Therefore, our study did not perform Y-randomisation tests, which is where our paper is flawed, and this would have some impact on the robustness of our model.

Finally, the models developed within this study can be utilized for predicting the behavior of PAEs upon interaction with cells as well as to the design of new, less toxic PAEs in the future.

## 4. Conclusions

In this paper, in vitro experiments found that PAEs after 72 h can inhibit the viability of the HepG2 cells to varying degrees, and at the same time, because of their molecular differences, PAEs can increase or inhibit the content of Nrf2 protein. On this basis, this study established PAEs 3D-QSAR model for LD_50_, IC_50_ and Nrf2. The models (LD_50_ and Nrf2) built for prediction were relatively stable and had a good predictive effect. In terms of structure-effect analysis, it was found that the steric field and the hydrophobic field had the greatest impact, but the electric field also had a certain contribution value. The results show that the introduction of large and hydrophobic groups on the side and branched chains may significantly affect the toxic effects of PAEs on cells. The results observed in this study present a potential tool for predicting the LD_50_ and Nrf2 of new PAEs based on the structure of the compounds, and also provide a reference for the design of new less toxic PAEs in the future. However, the model in our article suffers from a lack of data, which is the key to the overall model not being as perfect as it could be. We hope to improve this issue in future experiments.

## Figures and Tables

**Table 1:** Actual value, predicted value and error of 3D-QSAR model of PAEs lgLD_50_.

Names	Structures	LD_50_ (mg/kg)	Exp. (lgLD_50_)	CoMFA Pred.	CoMFA Res.	CoMSIA Pred.	CoMSIA Res.
DMP	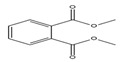	6800	3.8325	3.791	0.0415	3.829	0.0035
DEP	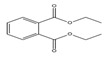	8600	3.9345	3.89	0.0445	3.942	−0.0075
DBP	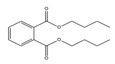	7499	3.875	3.955	−0.08	3.896	−0.021
DIBP	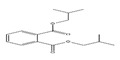	15,000	4.1761	4.241	−0.0649	4.175	0.0011
DHXP	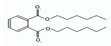	29,600	4.4713	4.417	0.0543	4.45	0.0213
DNOP	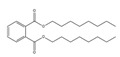	47,000	4.6721	4.647	0.0251	4.684	−0.0119
DIOP	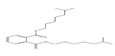	22,000	4.3424	4.374	−0.0316	4.339	0.0034
DEHP	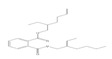	30,000	4.4771	4.475	0.0021	4.477	0.0001
DNP	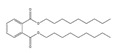	21,500	4.3324	4.458	−0.1256	4.332	0.0004
DIDP	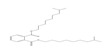	64,000	4.8062	4.759	0.0472	4.804	0.0022
nDNOP	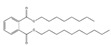	45,200	4.6551	4.604	0.0511	4.647	0.0081
DAP	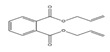	656	2.8169	2.821	−0.0041	2.815	0.0019
DMEP	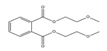	2750	3.4393	3.436	0.0033	3.419	0.0203

**Table 2:** 3D-QSAR models’ parameters.

3D-QSAR Model.	Styles	Q2	R2	F	SEE	S	E	H	D	A
LD_50_	CoMFA	0.522	0.991	0.067	192.025	58.4	41.6			
	CoMSIA1	0.621	1	0.015	1528.55	18.8	26.6	50.8	3.8	0
	CoMSIA2	0.631	1	0.015	1552.090	19.3	28.4	52.3		
IC_50_	CoMFA	0.69	0.98	0.021	48.513	79	21			
IC_50_	CoMSIA1	0.687	0.926	0.036	24.907	20.5	22.3	36.1	0	21.2
Nrf2	CoMSIA1	0.512	0.966	0.075	42.754	23.2	11.8	51.9	0	13.1

**Table 3:** Actual value, predicted value and error of 3D-QSAR model of PAEs 72 h-lgIC_50_.

Names	Structures	72 h-IC_50_ (μM)	Exp. (lgIC_50_)	CoMFA Pred.	CoMFA Res.	CoMSIA Pred.	CoMSIA Res.
DEHP	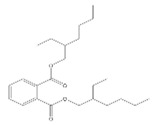	1160	3.0645	3.053	0.0115	3.069	−0.0045
DNP	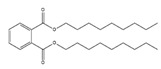	1513	3.18	3.206	−0.026	3.2	0.025
DIDP	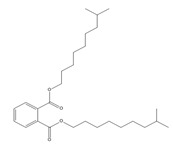	1722	3.236	3.232	0.004	3.094	−0.0052
DMEP	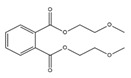	975.1	2.989	2.984	0.005	2.992	−0.003
DHXP	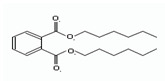	1227	3.0888	3.091	−0.0022	2.972	0.008
DNOP	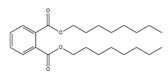	1678	3.225	3.206	0.019	3.233	0.003
DPHP	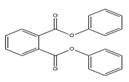	954.9	2.98	2.992	−0.012	3.203	−0.023

**Table 4:** Actual value, predicted value and error of 3D-QSAR model of PAEs on cell Nrf2 content.

Names	Structures	Exp. (Nrf2)	CoMSIA Pred.	CoMSIA Res.
DEHP	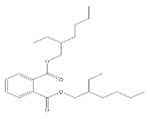	0.6449	0.646	−0.0011
DEP	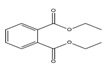	1.2220	1.140	0.082
DMP	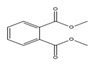	1.1534	1.151	0.0024
DMEP	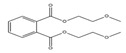	1.1691	1.170	−0.0009
DHXP	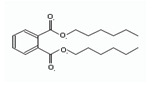	0.6882	0.673	0.0152
DNOP	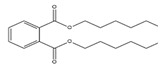	0.7129	0.720	−0.0071
DBP	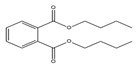	0.7363	0.746	−0.0097

## Data Availability

Data presented in this study are available on request from the Authors.
